# Impact of Image Enhancement Module for Analysis of Mammogram Images for Diagnostics of Breast Cancer

**DOI:** 10.3390/s22051868

**Published:** 2022-02-26

**Authors:** Yassir Edrees Almalki, Toufique Ahmed Soomro, Muhammad Irfan, Sharifa Khalid Alduraibi, Ahmed Ali

**Affiliations:** 1 Department of Medicine, Division of Radiology, Medical College, Najran University, Najran 61441, Saudi Arabia; 2 Department of Electronic Engineering, Larkana Campus, Quaid-e-Awam University of Engineering, Science and Technology, Nawabshah 67450, Pakistan; etoufique@yahoo.com; 3 Electrical Engineering Department, College of Engineering, Najran University, Najran 61441, Saudi Arabia; miditta@nu.edu.sa; 4 Department of Radiology, College of Medicine, Qassim University, Buraidah 52571, Saudi Arabia; salduraibi@qu.edu.sa; 5 Eletrical Engineering Department, Sukkur IBA University, Sukkur 65200, Pakistan; ahmedali.shah@iba-suk.edu.pk

**Keywords:** breast cancer, image enhancement, mammograph images, image segmentation, morphological operations, principal component analysis (PCA), seed-based region growth

## Abstract

Breast cancer is widespread around the world and can be cured if diagnosed at an early stage. Digital mammograms are used as the most effective imaging modalities for the diagnosis of breast cancer. However, mammography images suffer from low contrast, background noise as well as contrast as non-coherency among the regions, and these factors makes breast cancer diagnosis challenging. These problems can be overcome by using a new image enhancement technique. The objective of this research work is to enhance mammography images to improve the overall process of segmentation and classification of breast cancer diagnosis. We proposed the image enhancement for mammogram images, as well as the ablation of the pectoral muscle. The image enhancement technique involves several steps. In the first step, we process the mammography images in three channels (red, green and blue), the second step is based on the uniformity of the background on morphological operations, and the third step is to obtain a well-contrasted image using principal component analysis (PCA). The fourth step is based on the removal of the pectoral muscle using a seed-based region growth technique, and the last step contains the coherence of the different regions of the image using a second order Gaussian Laplacian (LoG) and an oriented diffusion filter to obtain a much-improved contrast image. The proposed image enhancement technique is tested with our data collected from different hospitals in Qassim health cluster Qassim province Saudi Arabia, and it contains the five Breast Imaging and Reporting System (BI-RADS) categories and this database contained 11,194 images (the images contain carnio-caudal (CC) view and mediolateral oblique(MLO) view of mammography images), and we used approximately 700 images to validate our database. We have achieved improved performance in terms of peak signal-to-noise ratio, contrast, and effective measurement of enhancement (EME) as well as our proposed image enhancement technique outperforms existing image enhancement methods. This performance of our proposed method demonstrates the ability to improve the diagnostic performance of the computerized breast cancer detection method.

## 1. Introduction

Breast cancer is one of the most invasive and deadly cancers in women around the world. According to the World Health Organization (WHO), 2.3 million women will have breast cancer in 2020, and there have been 685,000 deaths worldwide from breast cancer [1]. According to statistics, 7.8 million women have been living with breast cancer in the past five years, and it has classified breast cancer as the deadliest cancer in the world [2]. Breast cancer occurs in all countries of the world in women at any age after puberty, but it will increase later in life [3]. Breast cancer is the most common disease among Saudi women, with a prevalence of 21.8% [4]. A cancer survey has shown that breast cancer is one of the ninth leading causes of death among Saudi women [2]. In China, breast cancer is becoming the most common type of cancer diagnosed in women, accounting for 12.2% of global cases and 9.6% of all deaths worldwide.

Mammogram images are scanned for analysis of breast cancer, and it plays an essential role in detecting breast cancer early and helps reduce the death rate. But many factors affect the viewing of mammography images, and it is difficult for the radiologist to make a proper diagnosis [5]. These factors are breast tissue density, and mammography quality because the effectiveness of mammography ranges from 60 to 90%, and radiologists can only observe 10 to 30% of all mammographic lesions [6,7,8].

BI-RADS (Breast Imaging Reporting and Data System) is the mammographic standardization and quality assurance lexicon report represented by the American College of Radiology (ACR). The main objective of BI-RADS is to organize mammography report among radiologists and make it homogenize for clinicians [9]. BI-RADS mammography classifies qualitative characteristics and characterizes the shape, margin, and density of the mass of breast tissue. Radiologists categorized the mass in BI-RADS according to the defined features, as explained in Table 1.

Depending on the biological structure of the breast, cancer cells spread to the lymph nodes and affect other parts of the body, such as the lungs. Many researchers also found that hormonal lifestyle and environmental changes also lead to increased breast cancer [10]. The breast’s internal structures are obtained from a low dose X-ray of the breast, and this process is called mammography in biological terminology. Mammography is one of the most necessary methods of observing breast cancer because mammography radiates much lower radiation doses than other devices previously used. Recently, it has proven to be the safest modality for breast cancer screening [5].

There are two factors for the detection of breast cancer at an early stage: the proper acquisition process to obtain mammogram images and, secondly, accurate analysis of the mammogram images for the diagnosis of breast cancer. The manual process takes time and could delay the processing process. Accurate viewing of mammogram images is always a difficult task, especially with a large number of databases. This challenge can be overcome by using a computational method such as image processing techniques or the breast cancer analysis algorithm. These algorithms lead to rapid analysis and reduce the workload of medical experts. However, it is essential to study the nature of mammography images before using the algorithm based on the image process to detect breast cancer [11].

**Figure 1:**
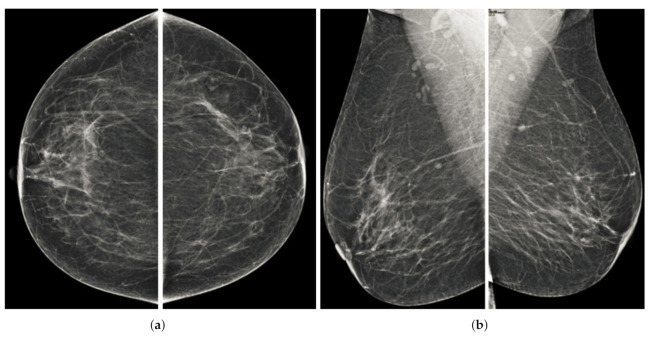
Representation of standard mammography views. The figure (**a**) represents the CC view of the right and left breast, and the figure (**b**) represents the MLO view of the right and left breast.

Computerized analysis of mammography is also challenging because one of the most important challenges is observing the pectoral muscles. The geometric shape of the pectoral muscles and their location depend on the mammographic images’ specific view [12,13]. There are two types of mammographic image views: the carnio-caudal (CC) view and the mediolateral oblique (MLO) view, and these views are shown in the Figure 1. The pectoral muscle on the CC view is semi-elliptical along the breast wall. In contrast, the MLO view covers most of the upper mammogram coverage and roughly corresponds to the overlapping right-angled triangle, as shown in Figure 1. Due to their appearance, both views suffered from low contrast, which made it difficult to see cancerous areas in some cases. Image enhancement requires correct observation and helps segment abnormal regions for disease classification. The quality of mammography images in terms of noise reduction and contrast enhancement is improved by using the image enhancement technique. The main purpose of implementing the image enhancement technique is to help the computerized breast cancer detection system to detect mammographic lesions with poor visibility and improve low contrast. Low contrast regions with small abnormalities are mostly hidden in the tissue of mammogram images, which makes it challenging to analyze the abnormal region, and also provides false detection.

Image enhancement techniques are generally divided into three categories. These categories are spatial domain, frequency domain and a combination of these two [14]. Further, these category-based image enhancement techniques are characterized into four types based on their use on the medical image and the nature of the image, namely conventional image enhancement techniques, region-based, feature-based, and fuzzy-based [15]. Conventional techniques are mainly used because they can be adapted for local as well as global enhancement of mammograms. Conventional techniques have the ability to improve the noise figure and enhance the image. Region-based image enhancement methods are best suited for contrast enhancement of a specified region with varying shape and size, but sometimes they give artifacts that lead to misclassification. Feature-based enhancement techniques are used for calcification on mammograms as well as their masses. Most transform-based techniques are used for enhancements of mammogram images, but the high frequencies remained, and this resulted in noise. Fuzzy enhancement is also used to normalize the image and give good contrast output, but it has also lost detail in service cases of mammography images.

In this research work, we proposed an image enhancement technique for mammography images for early detection of breast cancer. The image enhancement technique we propose has four steps. In the first and second steps, we will process the image and categorize it into three channels as mammography images are in jpeg format in almost all databases. We remove uneven illumination using morphological techniques to achieve a uniform background image. The third step contains obtaining a well-contrasted or grayscale image that gives us a good breast image with more observation of the abnormal region. The fourth step is to remove the pectoral muscle from the mammogram images, as the mammography images contain a lot of the background. We used a technique based on the seeds region points of the image depending on the orientation of the muscle. This four-step module is known as the Breast Cancer Image Enhancement Technique, and it gives us a computerized process of early detection of breast cancer. However, the main challenging task is the impact of the image enhancement technique on the segmentation and of the abnormal region. We have proposed a post-processing module based on second-order Gaussian filtering, diffusion filtering, and K-means for the segmentation of cancer regions to detect breast cancer. The main contributions of this research work are:

1. Implementation of novel image enhancement techniques for mammogram images and improves the overall quality of appearance.
2. This image enhancement technique can be used with post-processing steps for the early detection of breast cancer.
3. The contrast analysis for observation of abnormal lesions improves the segmentation and helps to diagnose the progress of breast cancer.
4. These pre-processing steps have improved the performance of existing methods based on machine learning.
5. The image enhancement may improve the training processing of an extensive database in supervised methods for breast cancer detection.

The article is organized as follows. Section 2 summarizes the related work. Section 3 explains in detail the proposed method and each step of the proposed method. Section 4 deals with databases and measurement parameters. Section 5 reports the experimental results. Finally, the conclusion and future directions are presented in Section 6.

## 2. Related Work

Mendez et al. [16] implemented the method based on filtering techniques to detect breast cancers from original mammogram images. They used the spatial averaging filter to smooth the image and the histogram-based thresholding, and after achieving the filtered image. They applied a gradient to track the abnormal region. Abdel et al. [17] proposed a technique based on different threshold values. Mario et al. [18] implemented the method based on wavelet decomposition and depth reduction of the background of mammograms images to get the cancerous area. They achieved 85% accuracy in their process. Karssemeijer and Brake [19] implemented a multiresolution scheme based on Hough transform to predicate the pectoral muscle accurately. Camillus et al. [20] developed a graph-cut-based breast segmentation method to identify the pectoral muscle. The result of their proposed method produced ragged lines with corrected using of Bezier curve. Ferrari et al. [21] modified the method proposed by Karssemeijer and Brake to detect the pectoral muscle. Kwok et al. [22] implemented the method for the detection of pectoral muscle by using the iterative threshold method. Wirth and Stapinski [23] implemented the active contour based method for breast region segmentation in mammographic images. Kwok et al. [24] implemented the method of computerized segmentation of pectoral muscles on a mediolateral oblique view of mammography images. They used the basic geometry concept, whereby the pectoral edge was estimated using a straight line and validated based on the location and orientation of mammography images. They removed the noise using the median filter, but their method still gave a false detection. Ferrari et al. [25] implemented the breast boundary identification method in mammography images. The method contained the modification of the contrast of the image, and they used the chain code algorithm for binarization. Next, they used the approximate contour as input to an active contour model algorithm to obtain the breast boundary for breast cancer analysis.

Raba et al. [26] developed the method based on an adaptive histogram to achieve the breast region from the background image. Then, the region growing-based method was used to get the pectoral muscle. Raba et al. [26] proposed the mammography segmentation technique in the breast region and background with pectoral muscle removal. They used an adaptive histogram approach to separate the breast from the background, then after using the region growing algorithm to remove the pectoral muscle to obtain a mammogram image. They tested 320 images and achieved around 98% accuracy, but achieved around 86% pectoral muscle suppression, and their method resulted in over-segmentation in dense image cases. Mirzaalian et al. [27] implemented a non-linear diffusion algorithm to remove the pectoral muscle. Martin et al. [28] implemented a method to obtain breast skin line in mammographic images. The edge of the breast provided the important information about the shape of the breast, as well as the deformation, which is generally used by image processing techniques and used image registration and anomaly detection. Kinsosita et al. [29] implemented a method using radon’s transform to estimate the pectoral muscle boundaries. Wang et al. [30] implemented the automated pectoral muscle detection based on discrete-time Markov chain and active contour model. Hu et al. [31] implementation a method for suspicious lesion segmentation in mammography images based on adaptive thresholding contained in multiresolution analysis. Their thresholding method based on global and adaptive local thresholding segmentation method and test on 170 images of mammograms, but then gave noise after using a morphological filter to remove noise, but still lost details and gave false detections. Chakraborty et al. [32] implemented a shape-based function with an average gradient to detect the position of the pectoral muscle boundary as a straight line. Chen and Zwiggelaar [33] implemented another histogram-based thresholding method to separate the breast area from the background. Connected components were used for the algorithm for labeling the segmented binary image of the abnormal region. After that, we used a region-based technique to remove the pectoral muscle, starting at a seed point closer to the pectoral muscle boundary.

Maitra et al. [34] implemented the method based on a triangular region to isolated the pectoral muscle from the rest of the tissue. Then, a region growing technique was used to remove the pectoral muscle. All these methods used either thresholding, region growing, or seed point image techniques. These techniques did not give us a good performance because the uniformity of image is required. However, image contrast enhancement techniques have been used for videos and images for decades to make image details more observable. Peng et al. [35] implemented the mammography image processing pipeline, which estimated the skin–air boundary using the gradient weight map to detect the breast pectoral boundary by adopting the method of unsupervised pixel labeling, and the final step was based on detection of the breast region with use of the texture filter. Bena et al. [36] implemented the mammography image segmentation method based on watershed segmentation and classification using K-NN classifier. The output is based on the grayscale co-occurrence matrices based on the Halarick texture function, and it was extracted from 60 mammography images. Kaitouni et al. [37] implemented breast tumor segmentation by pectoral muscle removal based on hidden Markov and region growth method. The purpose of the method was to separate the pectoral muscle from the mammographic images and to extract the breast tumor. The method contains two phases: Otsu thresholding and k-means based on image classification. Podgornova et al. [38] carried out the comparative study of methods for segmenting microcalcifications on mammography images. They used the watershed, mean shift, and k-means techniques in their comparative study, and the detection of k-means was comparable to the watershed and mean shift method, but yielded about 57.2% of false detections.

A few contrast enhancement techniques are useful for mammogram images to improve the performance of segmentation method, but most convention enhancement techniques are not useful for enhancing the contrast of mammogram images. In the last 21 years, many enhancement techniques have been implemented to enhance the low and varying contrast of mammogram images. Many detailed review papers are presented based on contrast enhancement techniques for mammogram images. Cheng et al. [39] discussed the conventional methods to features-based contrast enhancement techniques and their advantages and disadvantages. Stojic et al. [40] implemented mammogram images based on local contrast enhancement techniques and background noise removal. An improved method based on histograms-based contrast enhancement technique for X-ray images was implemented by Ming et al. [41]. Jianmin et al. [42] implemented an approach based on structure tensor and fuzzy enhancement operators for contrast enhancement of mammogram images. After reviewing all the methods for early detection of breast cancer, it was observed that a new enhancement technique is required to be used as a pre-processing module for breast cancer segmentation. We propose an image enhancement technique based on contrast sensitivity techniques in this research work for the early detection of breast cancer.

## 3. Proposed Method

**Figure 2:**
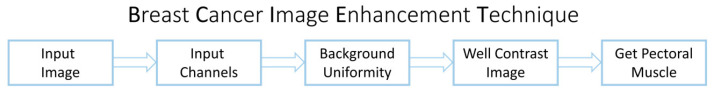
Our proposed breast cancer image enhancement technique.

Our proposed image contrast enhancement technique is shown in Figure 2. It contains four steps: Image acquisition process, background uniformity, archived the pectoral muscle, and obtained well contrast breast image. Each step is explained below.

### 3.1. Image Acquisition Process

**Figure 3:**
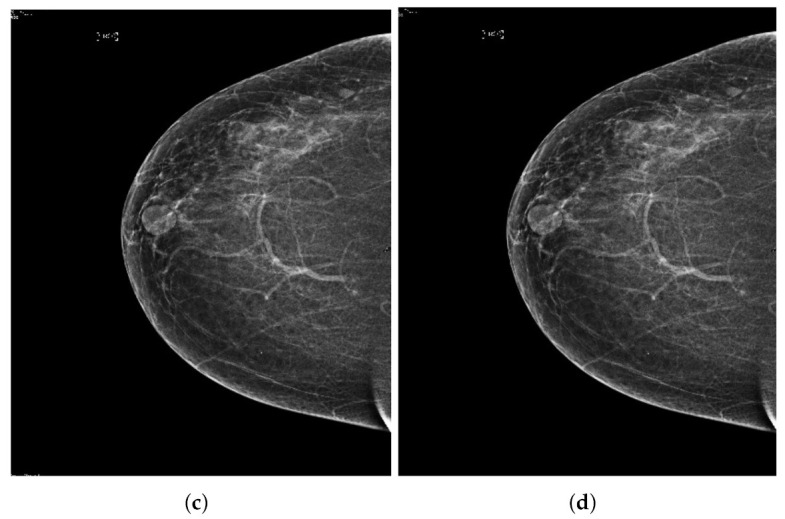

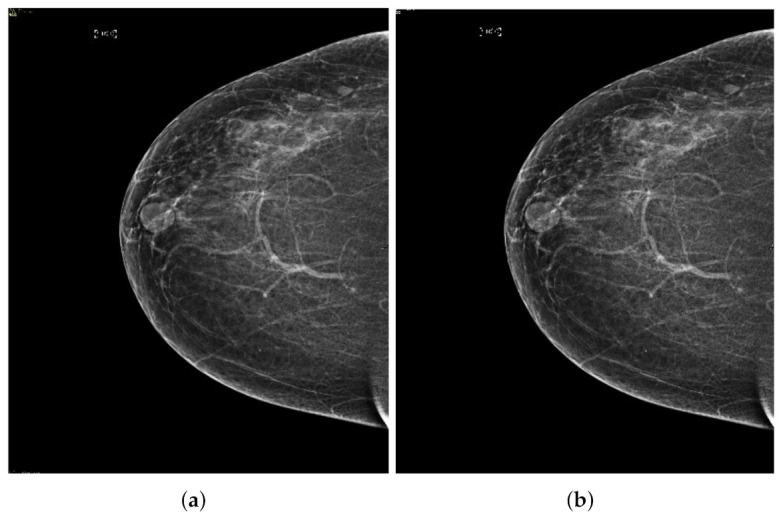
Processing of the mammogram images. (**a**) Input mammograph image processing. (**b**) Image of the mammograph image in the red channel. (**c**) Image of the mammograph image in the green channel. (**d**) Image of the mammograph image in the blue channel.

Mammogram images are processed as input images in our image enhancement technique to obtain the much-enhanced image. There are two types of mammography: film screen mammography and digital mammography. We used digital mammography, also known as full field digital mammography (FFDM) [43]. These digital mammography images are in JPEG format, so we converted the image to three channels (red, green and blue) as shown in the Figure 3. Each channel has different properties, so we will deal with each channel for background uniformity in the next step. Our main goal is to manage these mammography channels and obtain a well-contrasted grayscale output image for further processing in detectors for observations of cancerous regions. We use grayscale because it takes less processing time. The next step is to remove the irregular illuminations from the image. There is a detailed analysis of each channel of mammography images because the mammography modalities gave the images in X-ray format so that each channel looks like the original images. However, this is not the point because each channel has different imaging properties, making some details more observable and some lost. The red and blue channels contain a little more noise than the green channel. We need uniformity between each channel to obtain well-contrasted mammography images for further processing in post-processing for breast cancer diagnosis.

### 3.2. Background Uniformity

**Figure 4:**
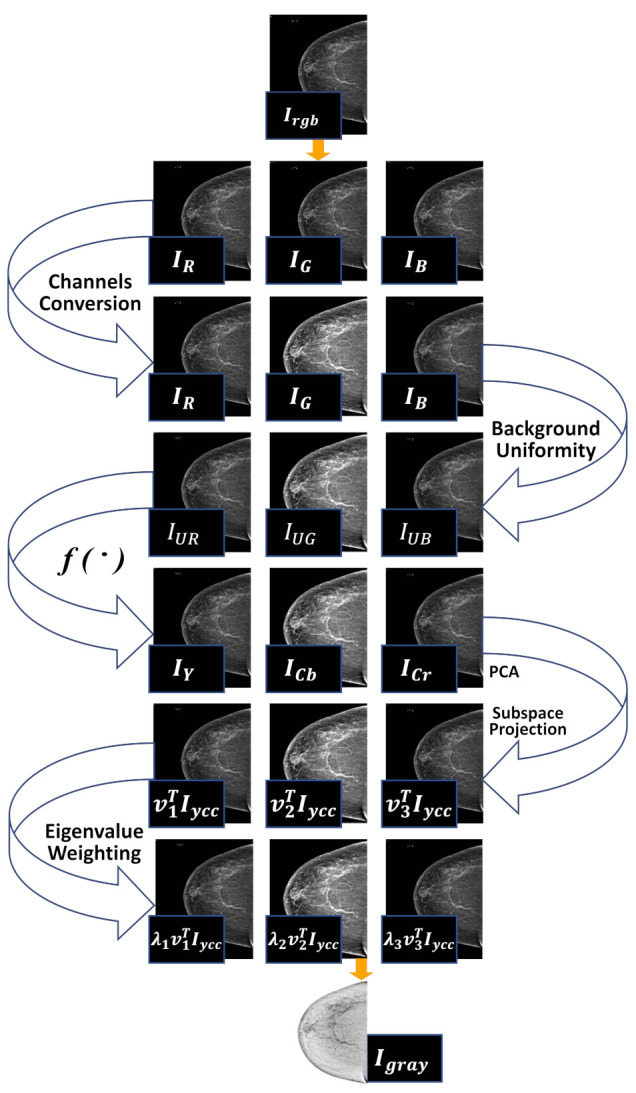
Overall conversion process PCA based color-to-gray conversion. Resulting well contrast mammogram image is obtained by using this PCA technique.

We process all three channels of mammography images. From our first step of the analysis, the green channel gives better contrast and more detail on the pectoral muscle than the other two channels. It has better histogram images that show good contrast distribution. However, there is still noise and uneven illumination due to the image acquisition process. Still, we cannot eliminate the analysis of the other two channels because we convert the three channels into a single grayscale image using the principal component analysis. It is the next step after removing uneven illumination and noise problem. We dealt with uneven illumination and noise using morphological techniques. We used basic morphological tactics called bottom hat and top hat operations to remove background noise and make the image contrast uniform. It is observed that there are variations of intensities in the image, and especially the region of the pectoral muscles, as shown in Figure 4. Because the intensity level of the pectoral muscle varies significantly due to an abnormal region or cancerous region, and these are significantly lower than the background intensity. Scanning the cancerous area is critical because of tiny nerves or vessels. The morphological bottom hat improves image analysis of such a region and provides more information to the image while lowering the noise level and observing the cancerous region. The Equation (1) shows the mathematical form of the bottom-hat operation.

(1) $$T_{b}\left(f\right)=f•b-f.$$

The • shows the close operation. Then, we applied the top hat operation to increase the contrast and control the change in contrast of the pectoral muscle or the cancerous region. The mathematical representation of the top hat operation is defined in the Equation (2). The ∘ shows the opening operation. An improved image is obtained with uneven illumination and noise suppression, but an appropriate grayscale image is still required. We used principal component analysis in the following steps to obtain a well-contrasted mammography image.

(2) $$T_{w}\left(f\right)=f-f\circ b.$$

### 3.3. Conversion into Grayscale Well Contrasted Image

After the background uniformity of the channels and eliminating most of the non-uniform regions of the image, the next most important task is to combine all the channels into a single grayscale image. It is necessary to remove the amount of data to be processed by the later stages of breast cancer detection. Many types of research have selected the green channel, instead of selecting the green channel only for its possible grayscale representation as adopted by many earlier researchers in this area [44], we prefer to use all three channels with the help of principal component analysis (PCA) to obtain a grayscale image.

Color-to-gray conversion is adopted to combine all previously processed tricolor images with their respective non-uniform removal process. We used the PCA technique for converting three channels to a grayscale image. The first step involves forming a vector color image ($I_{rgb}\varepsilon R^{3\times n}$) by stacking three channels side by side. Then, a $I_{YCbCr}$ image ($I_{YCC}\in R^{3\times n}$) is calculated from its original image to separate the luminance and chrominance channels using the conventional transfer function $f\left(•\right)$ as defined in [45]. In the next step, the eigenvalues $\lambda_{1}\geq \lambda_{2}\geq \lambda_{3}\in R^{1}$ and their corresponding eigenvectors $v_{1},v_{2},v_{3}\in R^{3}$ are projected by adopting the method of principal component analysis (PCA). The final gray image $I_{gray}\in R^{n}$ is calculated by a weighted linear combination of three projections, where the weights are calculated as a percentage of their eigenvalues. The final output is scaled to [0,255] as shown in the Figure 4. Next, we use the first subspace projection, which dominates the color-gray mapping results due to its substantially larger eigenvalue. However, the second and third subspace projections contribute a small proportion to the detail of the original three-channel image in the resulting grayscale image, as shown in Figure 4.

### 3.4. Removal of Pectoral Muscle

**Figure 5:**
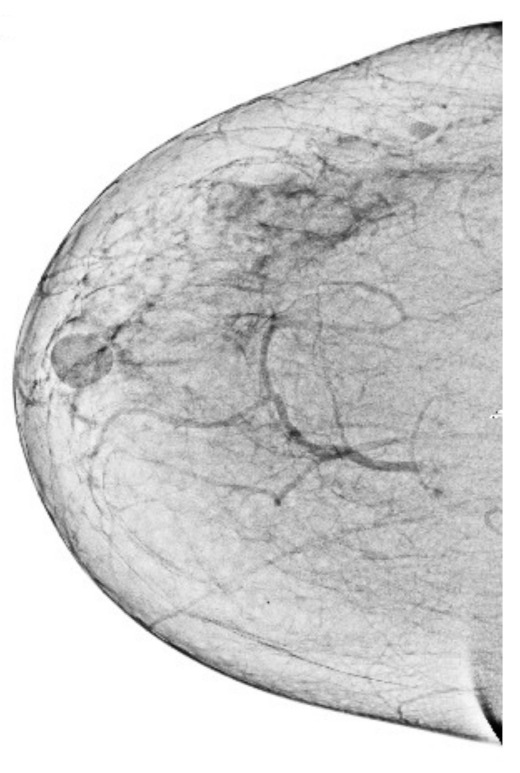
Final breast image after removal of pectoral muscle.

After getting a grayscale image, the next step is to reduce the pectoral muscle, as shown in Figure 5. We used the seed-based region growth technique to shrink the pectoral muscle to get the breast part. The breast is the region of interest in the mammogram images, and the breast contains the cancerous region. The seed region growing is one method of image segmentation, and it contains two operating principles. One principle is based on selecting the pixel location value and the other principle is selecting the seed point. We used the seed point method because it automatically gives an accurate selection based on the orientation of the image. In our case, the seed point is automatically selected considering the orientation of the mammographic image. The seed points are obtained by using the neighboring pixels of the seed and determining whether subsequent pixels should be added to the region or not. This process is continuous and iterative until the segmentation of the region of interest. The output of the seed region growing technique is shown in the Figure 5, and the breast region is observed to be affected. It facilitates obtaining abnormal region or cancerous region for segmentation of abnormal region, which leads to the higher classification accuracy of breast cancer detection. It is briefly explained in the Section 5.

### 3.5. Coherence of the Abnormal Regions

The main goal of breast cancer analysis from mammography images is to detect the abnormal or cancerous region as much as is precise. Many researchers did not consider the coherency of the abnormal region or mammogram images because they focused on the segmentation of the abnormal region. However, we have noticed that the coherency of mammography images increases visualization and facilitates segmentation of abnormal regions, leading to the classification of breast cancers. In this article, we consider the consistency of mammography images.

**Figure 6:**
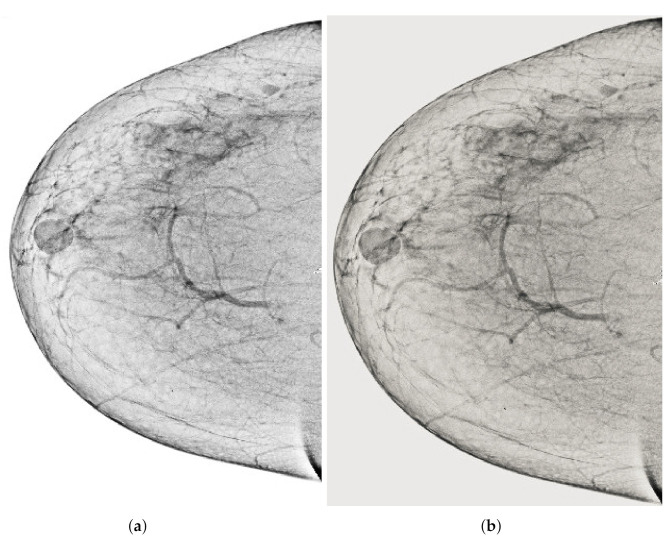
Mammogram coherent images. (**a**) Second order Laplacian of Gaussian output image and (**b**) anisotropic oriented diffusion filter image.

Mammographic image coherency contains two steps, the first step is based on the second-order Gaussian Laplacian (LoG) to normalize the set of mammography images as the output is shown in Figure 6. Although some regions, especially the regions containing a breast mass, the pixels are low intensities. These low pixels areas are not observed correctly, making it difficult to segment the cancerous area. We used oriented diffusion filtering as a second step to obtain a well-normalized image.

The more coherent images lead to more precise segmentation of the cancerous region from the mammography images. We apply the oriented diffusion filter [46] for coherent areas of low contrast region, as it is a suitable filtering method for normalized regions with low contrast. The oriented diffusion filter requires the recomputed orientation data of the image in advance, and this orientation data is called the orientation field (OF). The orientation field makes the diffusion tensor with the flow of the pixels of the image. The primary motivation for using an anisotropic diffusion procedure is based on the tilt angle of the best ellipse. It is achieved through the Second Order Gaussian detector, which gives the right direction for the low contrast region. The diffusion procedure is defined as follows:

1. Calculate the second moment matrix for each pixel.
2. Create the diffusion matrix for each pixel.
3. Calculate the change in intensity for each pixel as $\nabla \left(D\nabla I\right)$, where *D* is 2×2 diffusion matrix and *I* is the image entered in the process.
4. Update the image using the diffusion equation as follows:
  (3) $$I^{t+△t}=I^{t}+△t\times \nabla \left(D\nabla I\right).$$

The diffusion process is an iterative algorithm that processes the pixels from the initial mammography images to develop a smoother structure with each step [47]. The fine structure is achieved with a normalized image; there should be an appropriate stop criterion to get a fine structure. The stopping criterion is introduced in recent research works. The stop iteration process of the stop criterion is based on the rate of change of the spatial entropy value of the image relative to the number of iterations. The Figure 6a shows the output of the anisotropic oriented diffusion filter, and the output of the second-order Gaussian filter is also shown in the Figure 6b. It observed that this anisotropic oriented diffusion filter gives more coherence to the images in particular area, especially the area of the breast concerning the background compared to the Gaussian second order filter.

## 4. Database and Measuring Parameters

### 4.1. Databases

#### 4.1.1. Qassim Health Cluster Database

We collected the data from different hospitals in Qassim health cluster, Qassim province, Saudi Arabia hospital, and the database contains 11,194 mammogram images, and the database is categorized according to the Breast Imaging Data and Reporting System (BI-RADS). The number of images in the database is shown in the Table 2. Still, we used 100 images from each category of BI-RADS and 100 images from negative cases or BI-RADS-1 because our goal is to validate the image enhancement technique to be used as a pre-processing step for the classification of breast cancer.

#### 4.1.2. Mammographic Image Analysis Society (MIAS) Database

The Mammographic Image Analysis Society (MIAS) was created by British research groups with the aim of understanding images from mammograms. Mammography images are from the UK National Breast Screening Programme. The images are digitized at 50 microns of pixel edge with a Joyce-Loebl scanning microdensitometer, a linear device in the 0–3.2 optical density range and representing each pixel with an 8-bit word. The MIAS database contains 322 digitized images of 161 pairs. The database has been reduced to a 200 micron pixel edge and padded so that all images are 1024×1024.

### 4.2. Measuring Parameters

The proposed image enhancement technique is evaluated based on contrast enhancement, noise reduction and unwanted artifacts, and more details. We calculated the three parameters to know the effectiveness of our proposed image enhancement technique for mammography images:

(1) Peak signal to noise ratio (PSNR);
(2) Image contrast;
(3) Effective measure of enhancement (EME).

#### 4.2.1. Peak Signal to Noise Ratio (PSNR)

There are many processes to calculate PSNR, but we figured the value of PSNR based on the mean square estimation (MSE). First, we will calculate the MSE (as shown in the Equation (5)) of the image tracks to calculate the PSNR (as shown in the equation). The main objective of the PSNR is widely used to calculate the quality of images between the original image and the output image in terms of noise reduction. The large value of PSNR means that the image has less noise compared to the original image.

(4) $$MSE=\frac{1}{MN}\sum_{m}{\sum_{n}\left|X\left(m,n\right)-\left.Y\left(m,n\right)\right|\right.}^{2}.$$

(5) $$PSNR=10\log_{10}\left[\frac{\left(L-1\right)}{MSE}\right].$$

where $X\left(m,n\right)$ and $Y\left(m,n\right)$ are the gray level of input and output image at pixels positions of $\left(m,n\right)$. The *L* is the maximum pixels value of image, and it would be 255 of 8-bit pixels image $\left[L=2^{n}-1=2^{8}-1=256-1=255\right]$. PSNR is measured in decibel (dB) in unit.

#### 4.2.2. Image Contrast

The image contrast gives information about the contrast enhancement and is calculated using the Equation (6). Where *M* and *N* represent the width and height of the image, respectively. The higher the contrast value, the better the image information. Taking the logarithm of contrast is converting it to decibel unit (dB), as shown in the Equation (7).

(6) $$C_{contrast}=\frac{1}{MN}\sum_{m=1}^{M}\sum_{n=1}^{N}Y^{2}\left(m,n\right)-{\left|\left.\frac{1}{MN}\sum_{m=1}^{M}\sum_{n=1}^{N}Y\left(m,n\right)\right|\right.}^{2}.$$

(7) $$C_{contrast}=10\log_{10}C_{contrast}.$$

#### 4.2.3. Effective Measure of Enhancement (EME)

The EME is the quantitative measure of image enhancement, and it gives information about the contrast of each image block and is calculated using the Equation (8). Where $K_{1}$ and $K_{2}$ are the numbers of horizontal and vertical blocks in the enhanced image, $I_{\max}\left(k,l\right)$ and $I_{\min}\left(k,l\right)$ are the image blocks’ maximum and minimum pixel values.

(8) $$EME=\frac{1}{K_{1}K_{2}}\sum_{L=1}^{K_{2}}\sum_{K=1}^{K_{1}}20\log\left(\frac{I_{\max}\left(k,l\right)}{I_{\min}\left(k,l\right)}\right).$$

## 5. Experimental Results Analysis

### 5.1. Analysis of Proposed Image Enhancement Technique

**Figure 7:**
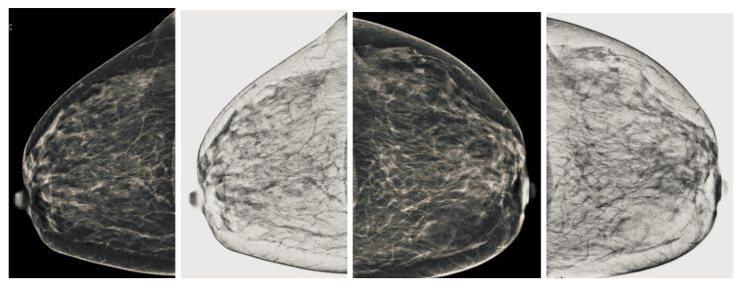
Analysis of CC view of BI-RADS-1 mammogram images.

**Figure 8:**
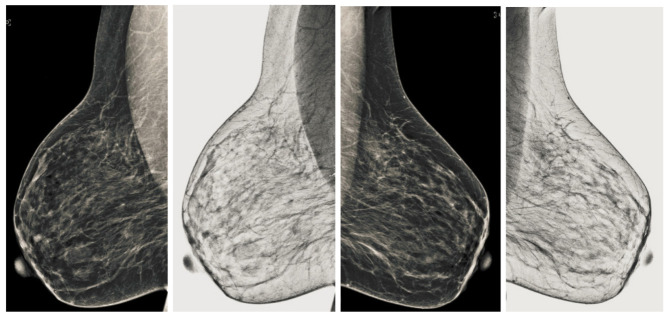
Analysis of MLO view of BI-RADS-1 mammogram images.

**Figure 9:**
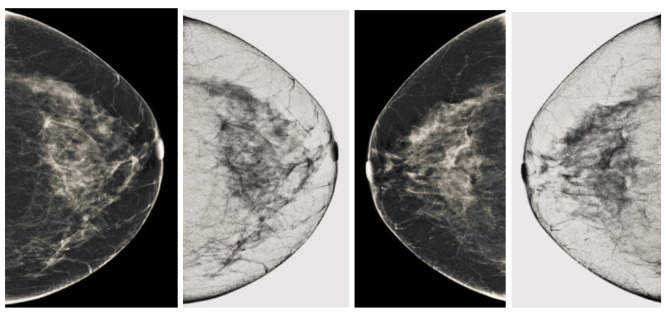
Analysis of CC view of BI-RADS-2 mammogram images.

**Figure 10:**
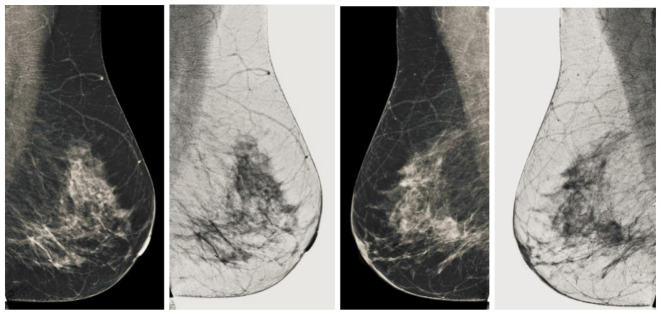
Analysis of MLO view of BI-RADS-2 mammogram images.

**Figure 11:**
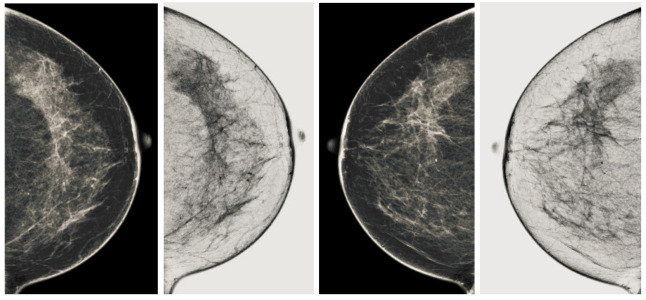
Analysis of CC view of BI-RADS-3 mammogram images.

**Figure 12:**
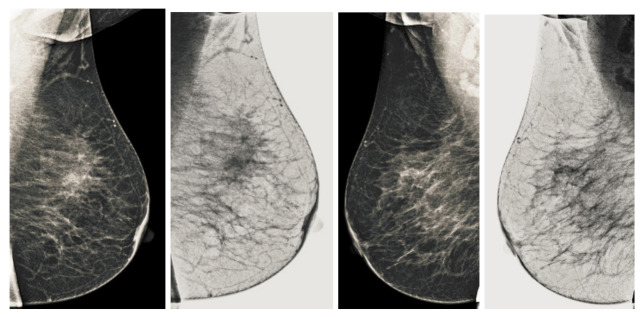
Analysis of MLO view of BI-RADS-3 mammogram images.

**Figure 13:**
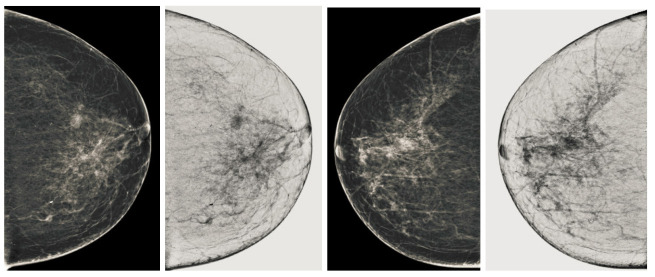
Analysis of CC view of BI-RADS-4 mammogram images.

**Figure 14:**
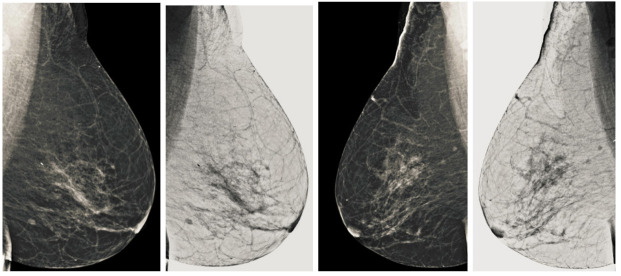
Analysis of MLO view of BI-RADS-4 mammogram images.

**Figure 15:**
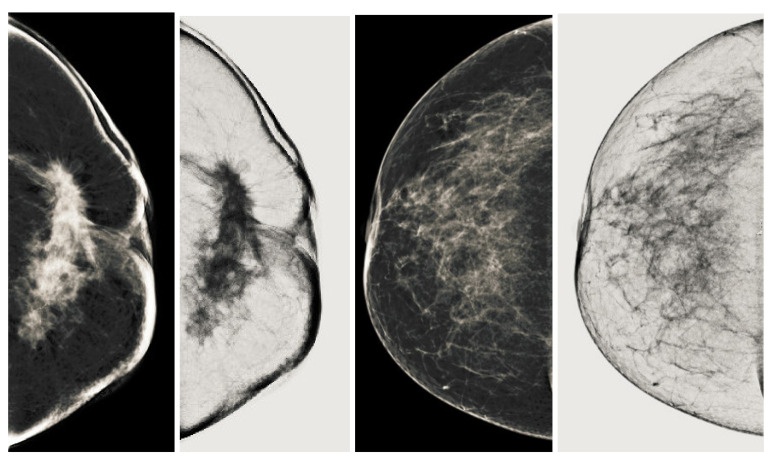
Analysis of CC view of BI-RADS-5 mammogram images.

**Figure 16:**
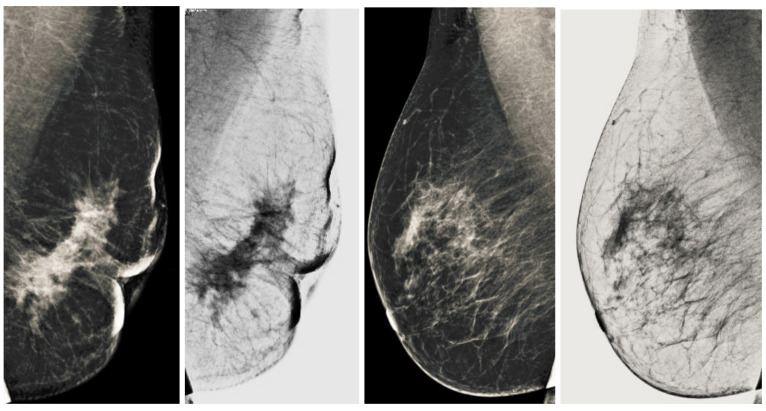
Analysis of MLO view of BI-RADS-5 mammogram images.

We also measured the PSNR, the image contrast, and the EME of each category of databases, as we analyzed the image in terms of visual observation. The Table 3 shows the performance of our proposed image enhancement method. It can be seen from the Table 3 that our proposed method improved PSNR, contrast, and EME, and this also shows that our method can work on every category of BI-RADS. Because many techniques do not work on higher grade BI-RADS due to the complexity and the images are not of good quality. We obtained an average improvement in PSNR, contrast, and EME in the Table 4. For more observations, we analyzed the visual image of each category and we analyzed the CC and MLO of each category as shown in the Figure 7, Figure 8, Figure 9, Figure 10, Figure 11, Figure 12, Figure 13, Figure 14, Figure 15 and Figure 16. From the figures, every detail of image of every category can be observed, leading to better segmentation of the abnormal region. This image enhancement technique can be used as preprocessing steps for the detection of breast cancer. It is a very fast processing algorithm and it takes on 21.13 s. It gives opportunity to medical experts to analyze the mammogram images very quickly to propose the timely treatment.

### 5.2. Comparatives Analysis of Image Enhancement with Existing Image Enhancement Techniques

We compare the performance of our proposed image enhancement technique with other techniques such as Histogram Equalization (HE), Contrast Limited Adaptive Histogram Equalization (CLAHE), Brightness Preserving Bi-Histogram Equalization (BBHE), Histogram Modified-Local Contrast Enhancement (HM-LCM), Bi-level Histogram Modification-Adaptive Nonlinear Filter (BHM-ANF) and Retinex. It can be observed from Table 5 and Table 6 that our proposed image enhancement technique outperforms other techniques in improving PSNR, contrast and EME. It shows the technique’s capacity for the analysis of mammography images for the diagnosis of breast cancer.

### 5.3. Impact of Image Enhancement Technique on Post-Processing

**Figure 17:**
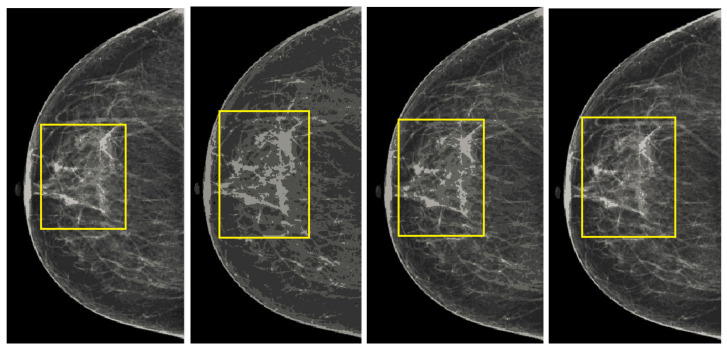
The output of our proposed post-processing step. The row shows output of Kmeans cluster 2, 4, 6 and 8.

We used the Kmeans as tested the capability of our proposed image enhancement technique. We have analyzed Kmeans with cluster parameters range from 0 to 8. We got the best output of Kmeans from cluster of 2, 4, 6 and 8. It is clearly observed in Figure 17 that abnormal region can easily seen from Kmeans output with yellow circle.

We measured the accuracy, sensitivity and specificity of our post-processing output as shown in the Table 7, and this yields considerable accuracy that shows the capability of our method. We used our own database created from Qassim Health Cluster, Qassim Province, Saudi Arabia Hospital. But we measured the ability of our proposed method by using the Mammographic Image Analysis Society (MIAS) database because many researchers have used these databases and measured the only Accuracy (AC) parameters, but we measured specificity (SP) and sensitivity (SE), and the comparison Table 8 shows that our method outperforms [23,24,25,28,33,34,37,38] terms of accuracy, but they did not report sensitivity and specificity. The method [31] reported sensitivity, but they did not report accuracy for evaluation. All these methods mainly used few images from the MIAS database, and their method based on active contour or threshold-based tactics, and the main limitation of these methods is lack of proper implementation of pre-processing steps or image enhancement technique according to nature of the mammogram images. As shown in Table 8, our method gave comparable performance with [26,35], but [26] reduced accuracy due to some cases of over segmentation, but their method can be improved by using new pre-processing steps for pectoral muscle removal. From the comparative performance of our proposed method with the existing method in Table 8 shows that our proposed pre-processing steps can play an important role for the implementation of fully automatic breast cancer screening, because the proposed image enhancement technique has shown the great impact on post-processing, which play an important role in deep learning based method, and this image enhancement technique can improve the performance of the data training process for the analysis of medical imaging methods.

## 6. Conclusions and Future Direction

The output image is considered to be an enhanced image compared to the original image by evaluation of the measurement parameters, as well as by visual observation. In this research work, the new image enhancement method is proposed and tested on all BI-RADS categories for the detection of breast cancer. The performance of the proposed method is analyzed on the basis of visual perception and quantitative measurements. We obtained the best enhanced image, and the performance of the proposed method is compared to state of the art contrast enhancement techniques such as HE, CLAHE and BBHE. Our proposed image enhancement technique is validated against a large database that contains approximately 11,000 images, but we have used approximately 700 images to validate this database. As in the basis on state-of-the-art, previous methods for breast cancer, whether enhancement-, segmentation-, or classification-based methods, did not use all categories of BI-RADS, but we have validated our technique from negative (BI-RADS-1) to malignant (BI-RADS-5) images.

Our proposed image enhancement technique contains the different steps to improve the contrast of the image in terms of visual perception, noise reduction as well as contrast enhancement. We used new imaging techniques to obtain a well-contrasted image as well as coherence filtering to obtain a well-normalized image. The main objective of the image enhancement technique is to aid the post processing steps of any image processing method or machine learning method. There is still room to improve the image enhancement technique as it gives an improved performance on all categories, but it decreases performance on the higher level of BI-RADS. Therefore, the tuning parameters-based filters like scaled normalized Gaussian filtering can be used to set parameters according to the properties of the image in order to obtain a well contrasted image. This image enhancement technique can also be used to improve the performance of a deep learning-based method for breast cancer, as it can be used as an input technique for the training process. Because good training of the database provides the best segmented output, and this is one of the contributions that we propose. This image enhancement technique can improve the training process of the machine learning method and it also improves the performance of traditional methods when used as a pre-treatment module.

## Figures and Tables

**Table 1:** Radiologists categorized the mass in BI-RADS.

Category	Remarks
00	Process is incomplete and it requires further assessment.
01	It is negative and there are no abnormities.
02	It is an initial stage (It is benign of breast cancer).
03	It is a mild stage (It is probably benign of breast cancer).
04	It is a moderate stage (It is suspicious of breast cancer).
05	It is a high malignancy stage (It is highly suspicious cancer).
06	It is the final stage and known biopsy-proven malignancy.

**Table 2:** Database information.

Category	Number of Images
BI-RADs-1	996
BI-RADs-2	817
BI-RADs-3	371
BI-RADs-4	452
BI-RADs-5	256

**Table 3:** Performance of proposed method on database basis on category wise.

	Without Image Enhancement			With Image Enhancement		
**Category of BI-RADS**	**PSNR**	**Contrast**	**EME**	**PSNR**	**Contrast**	**EME**
BI-RADs-1	28.13	45.13	5.12	32.48	71.87	8.16
BI-RADs-2	27.21	42.26	4.95	31.25	67.81	7.42
BI-RADs-3	26.05	39.98	4.31	30.01	65.23	6.81
BI-RADs-4	25.98	38.54	4.02	28.93	63.12	6.51
BI-RADs-5	25.54	37.98	3.98	27.57	62.01	5.91

**Table 4:** Performance improvement in PSNR, contrast and EME.

Category of BI-RADS	PSNR	Contrast	EME
BI-RADs-1	4.35	26.74	3.04
BI-RADs-2	4.04	25.55	2.47
BI-RADs-3	3.96	25.25	2.5
BI-RADs-4	2.95	24.58	2.49
BI-RADs-5	2.33	24.03	1.93

**Table 5:** Comparative analysis of proposed methods with existing image enhancement techniques. Note: Bold shows the performance of our proposed image enhancement technique.

Techniques	HE			CLAHE			BBHE			Proposed Method		
**Category**	**PSNR**	**Contrast**	**EME**	**PSNR**	**Contrast**	**EME**	**PSNR**	**Contrast**	**EME**	**PSNR**	**Contrast**	**EME**
BI-RADs-1	12.13	19.10	1.98	18.95	21.24	1.92	17.08	19.87	1.48	**32.48**	**71.87**	**8.16**
BI-RADs-2	11.28	17.15	1.25	17.34	20.05	1.55	15.21	18.91	1.35	**31.25**	**67.81**	**7.42**
BI-RADs-3	11.17	17.04	1.02	16.95	19.75	1.22	15.01	17.81	1.11	**30.01**	**65.23**	**6.81**
BI-RADs-4	10.91	16.13	1.01	16.28	18.92	1.18	16.19	18.01	1.12	**28.93**	**63.12**	**6.51**
BI-RADs-5	10.12	16.01	0.99	16.21	17.93	1.01	16.17	17.87	1.02	**27.57**	**62.01**	**5.91**

**Table 6:** Comparative analysis of proposed methods with existing image enhancement techniques. Note: Bold shows the performance of our proposed image enhancement technique.

Techniques	HM-LCM			BHM-ANF			Retinex			Proposed Method		
**Category**	**PSNR**	**Contrast**	**EME**	**PSNR**	**Contrast**	**EME**	**PSNR**	**Contrast**	**EME**	**PSNR**	**Contrast**	**EME**
BI-RADs-1	16.23	18.22	1.92	17.35	19.03	1.98	19.98	20.12	1.62	**32.48**	**71.87**	**8.16**
BI-RADs-2	14.38	19.35	1.55	18.84	22.01	1.67	16.34	19.72	1.43	**31.25**	**67.81**	**7.42**
BI-RADs-3	12.37	18.24	1.22	18.35	21.25	1.52	17.32	20.06	1.47	**30.01**	**65.23**	**6.81**
BI-RADs-4	11.71	17.27	1.02	17.63	20.64	1.08	17.07	20.31	1.43	**28.93**	**63.12**	**6.51**
BI-RADs-5	11.32	17.09	1.09	17.01	19.97	1.06	17.21	18.37	1.32	**27.57**	**62.01**	**5.91**

**Table 7:** Performance of proposed segmentation model on Qassim Health Cluster, Qassim Province, Saudi Arabia Hospital database.

Category of BI-RADS	Specificity	Sensitivity	Accuracy
BI-RADs-1	96.98	84.12	97.09
BI-RADs-2	96.18	83.14	96.89
BI-RADs-3	96.11	82.98	96.74
BI-RADs-4	95.18	82.83	95.12
BI-RADs-5	94.98	82.19	94.87

**Table 8:** Performance of proposed segmentation model on MIAS. Note: Bold shows the performance of our proposed Method.

Method	Images Used for Experiment	SP	SE	AC
Wirth & Stapinski [23]	25	-	-	97
Kwok et al. [24]	322	-	88	83.9
Ferrari et al. [25]	84	-	-	96
Raba et al. [26]	320	-	-	98
Marti et al. [28]	65	-	-	97
Hu et al. [31]	170	-	91.3	-
Chen & Zwiggelaar [33]	322	-	-	92.8
Maitra et al. [34]	322	-	-	95.7
Peng et al. [35]	322	-	-	97.08
Beena et al. [36]	60	-	-	83.33
Kaitouni et al. [37]	322	-	-	91.92
Podgornova et al. [38]	250	-	-	90.05
**Proposed Method**	**322**	**95.97**	**84.7**	**97.9**

## Data Availability

The data is available and could be shared upon request.

## References

[1] Ferlay J., Soerjomataram I., Dikshit R., Eser S., Mathers C., Rebelo M., Parkin D.M., Forman D., Bray F. (2015). Cancer incidence and mortality worldwide: Sources, methods and major patterns in globocan 2012. Int. J. Cancer.

[2] American Cancer Society (2018). Global Cancer: Facts and Figures.

[3] Fan L. (2014). Breast cancer in China. Lancet Oncol..

[4] DeSantis C.E., Ma J., Sauer A.G., Newman L.A., Jemal A. (2014). Breast cancer statistics, 2017, Racial disparity in mortality by state. CA Cancer J. Clin..

[5] Gupta S., Chyn P.F., Markey M.K. (2006). Breast cancer CADx based on BI-RADS™ descriptors from two mammographic views. Med. Phys..

[6] Verma B., McLeod P., Klevansky A. (2010). Classification of benign and malignant patterns in digital mammograms for the diagnosis of breast cancer. Expert Syst. Appl..

[7] Lévy L., Suissa M., Bokobsa J., Tristant H., Chiche J.-F., Martin B., Teman G. (2005). Presentation of the French translation of the Breast Imaging Reporting System and Data System (BI-RADS). Gynecol. Obstet. Fertil..

[8] Blakely T., Shaw C., Atkinson J., Cunningham R., Sarfati D. (2011). Social inequalities or inequities in cancer incidence? Repeated census-cancer cohort studies. New Zealand 1981–1986 to 2001–2004. Cancer Causes Control.

[9] Lee A.Y., Wisner D.J. (2017). Inter-reader variability in the use of BI-RADS descriptors for suspicious findings on diagnostic mammography: A multi-institution study of 10 academic radiologists. Acad. Radiol..

[10] Smigal C., Jemal A., Ward E., Cokkinides V., Smith R., Howe H.L., Thun M. (2006). Trends in breast cancer by race and ethnicity: Update. CA Cancer J. Clin..

[11] National Council on Radiation (2012). Guide to Mammography and Other Breast Imaging Procedures.

[12] Ponraj D.N., Jenifer M.E., Poongodi D.P., Manoharan J.S. (2011). A survey on the preprocessing techniques of mammogram for the detection of breast cancer. J. Emerg. Trends Comput. Inf. Sci..

[13] Rangayyan R.M., Ayres F.J., Leo Desautels J.E. (2007). A review of computer-aided diagnosis of breast cancer: Toward the detection of subtle signs. J. Frankl. Inst..

[14] Shih F.Y. (2010). Image Processing and Pattern Recognition: Fundamentals and Techniques.

[15] Biltawi M., Al-Najdawi N., Tedmori S. Mammogram Enhancement and Segmentation Methods: Classification, Analysis, and Evaluation. Proceedings of the 13th International Arab Conference on Information Technology.

[16] Mendez A., Tahoces P. (1996). Automatic detection of breast border and nipple in digital mammograms. Comput. Methods Programs Biomed..

[17] Abdel M.M., Carman C., Hills C.R., Vafai S. (1996). Locating the boundary between the breast skin edge and the background in digitized mammograms. Digital Mammography.

[18] Mario M., Jelena B., Mislav G. Breast border extraction and pectoral muscle detection using wavelet decomposition. Proceedings of the IEEE EUROCON.

[19] Karssemeijer N., Brake T.G. Combining single view features and asymmetry for detection of mass lesions. Proceedings of the 4th International Workshop Digital Mammography.

[20] Camilus K.S., Govindan V.K., Sathidevi P.S. (1998). Computer-aided identification of the pectoral muscle in digitized mammograms. J. Digit. Imaging.

[21] Ferrari R.J., Rangayyan R.M., Desautels J.E.L., Frere A.F. Segmentation of mammograms: Identification of the skin-air boundary, pectoral muscle, and fibroglandular disc. Proceedings of the 5th International Workshop on Digital Mammography.

[22] Kwok S.M., Chandrasekhar R., Attikiouzel Y. Automatic pectoral muscle segmentation on mammograms by straight line estimation and cliff detection. Proceedings of the 7th Australian and New Zealand Intelligent Information Systems Conference.

[23] Wirth M.A., Stapinski A. Segmentation of the breast region in mammograms using active contours. Proceedings of the SPIE 5150, Visual Communications and Image Processing.

[24] Kwok S.M., Chandrasekhar R., Attikiouzel Y., Rickard M.T. (2004). Automatic pectoral muscle segmentation on mediolateral oblique view mammograms. IEEE Trans. Med. Imaging.

[25] Ferrari R.J., Rangayyan R.M., Desautels J.E., Borges R.A., Frère A.F. (2004). Identification of the breast boundary in mammograms using active contour models. Med. Biol. Eng. Comput..

[26] Raba D., Oliver A., Martí J., Peracaula M., Espunya J. (2005). Breast segmentation with pectoral muscle suppression on digital mammograms. Lect. Notes Comput. Sci..

[27] Mirzaalian H., Ahmadzadeh M.R., Sadri S. Pectoral muscle segmentation on digital mammograms by nonlinear diffusion filtering. Proceedings of the Pacific Rim Conference on Communications.

[28] Marti R., Oliver A., Raba D., Freixenet J. Breast Skin-Line Segmentation Using Contour Growing. Proceedings of the Iberian Conference on Pattern Recognition and Image Analysis.

[29] Kinoshita S.K., Azevedo M.P.M., Pereira R.R., Rodrigues J.A.H., Rangayyan R.M. (2008). Radon-domain detection of the nipple and the pectoral muscle in mammograms. J. Digit. Imaging.

[30] Wang L., Zhu M., Deng L., Yuan X. (2010). Automatic pectoral muscle boundary detection in mammograms based on markov chain and active contour model. J. Zhejiang Univ. Sci. C.

[31] Hu K., Gao X., Li F. (2011). Detection of suspicious lesions by adaptive thresholding based on multiresolution analysis in mammograms. IEEE Trans. Instrum. Meas..

[32] Chakraborty J., Mukhopadhyay S., Singla V., Khandelwal N., Bhattacharyya P. (2011). Automatic detection of pectoral muscle using average gradient and shape based feature. J. Digit. Imaging.

[33] Chen Z., Zwiggelaar R. A combined method for automatic identification of the breast boundary in mammograms. Proceedings of the 5th International Conference on BioMedical Engineering and Informatics.

[34] Maitra I.K., Nag S., Bandyopadhyay S.K. (2012). Technique for preprocessing of digital mammogram. Comput. Methods Programs Biomed..

[35] Shi P., Zhong J., Rampun A., Wang H. (2018). A hierarchical pipeline for breast boundary segmentation and calcification detection in mammograms. Comput. Biol. Med..

[36] Beena Ullala Mata B.N., Meenakshi M. (2018). Mammogram image segmentation by watershed algorithm and classification through k-NN classifier. Bonfring Int. J. Adv. Image Process..

[37] Kaitouni S.E.I.E., Abbad A., Tairi H. (2018). A breast tumors segmentation and elimination of pectoral muscle based on hidden Markov and region growing. Multimed. Tools Appl..

[38] Podgornova Y.A., Sadykov S.S. (2019). Comparative analysis of segmentation algorithms for the allocation of microcalcifications on mammograms. Inf. Technol. Nanotechnol..

[39] Cheng H.D., Cai X., Chen X., Liming H., Xueling L. (2003). Computer-aided detection and classification of microcalcifications in mammograms: A survey. Pattern Recognit..

[40] Stojic T., Reljin I., Reljin B. Local contrast enhancement in digital mammography by using mathematical morphology. Proceedings of the International Symposium on Signals, Circuits and Systems (ISSCS 2005).

[41] Ming Z., Youfu L., Qinghao M., Ting Y., Jian L. (2012). Improving histogram-based image contrast enhancement using gray-level information histogram with application to X-ray images. Optik.

[42] Jianmin J., Bin Y., Wason A.M. (2005). Integration of fuzzy logic and structure tensor towards mammogram contrast enhancement histogram modification framework. Comput. Med. Imaging Graph..

[43] Prabhpreet K., Gurvinder S., Parminder K. (2019). Intellectual detection and validation of automated mammogram breast cancer images by multi-class SVM using deep learning classification. Inform. Med. Unlocked.

[44] Marin D., Aquino A., Gegundez-Arias M.E., Bravo J.M. (2011). A new supervised method for blood vessel segmentation in retinal images by using gray-level and moment invariants-based features. IEEE Trans. Med. Imaging.

[45] (1995). 601-5 IRB: Studio Encoding Parameters of Digital Television for Standard 4:3 and Wide Screen 16:9 Aspect Ratios. https://www.itu.int/rec/R-REC-BT.601/.

[46] Fehrenbach J., Mirebeau J.M. (2014). Sparse non-negative stencils for anisotropic diffusion. J. Math. Imaging Vis..

[47] Khan T.M., Khan M.A., Kong Y., Kittaneh O. (2016). Stopping criterion for linear anisotropic image diffusion: A fingerprint image enhancement case. EURASIP J. Image Video Process..

